# Reducing pesticide risks to farming communities: cotton farmer field schools in Mali

**DOI:** 10.1098/rstb.2012.0277

**Published:** 2014-04-05

**Authors:** William Settle, Mohamed Soumaré, Makhfousse Sarr, Mohamed Hama Garba, Anne-Sophie Poisot

**Affiliations:** 1 AGPM Division, United Nations Food and Agriculture Organization (UNFAO), Viale delle Terme di Caracalla, Rome 00153, Italy; 2 FAO Representation Avenue de la Liberté - Dar Salam, (Route de Koulouba) Commune 3 1820, Bamako, Mali; 3 FAO Representation 15 Rue Calmette, angle rue El Hadj Amadou Assane Ndoye 3300, Dakar, Senegal

**Keywords:** farmer field school, pesticides, adaptive management, cotton, integrated pest management, Africa

## Abstract

We provide results from a study of two separate sectors within the cotton-growing region of southern Mali. In one sector, farmers have engaged in a farmer field school (FFS) training programme since 2003—the other not. One goal of the training was the adoption of alternatives to the use of hazardous insecticides, through integrated pest management (IPM) methods. Over an 8-year period, analysis showed that with roughly 20% of the 4324 cotton-growing farm households having undergone training, hazardous insecticide use for the entire sector fell by 92.5% compared with earlier figures and with the second (control) sector. Yields for cotton in both sectors were highly variable over time, but no evidence was found for changes in yield owing to shifts in pest management practices. Evidence is presented for a likely diffusion of new practices having taken place, from FFS participants to non-participants. We discuss strengths and weaknesses of the FFS approach, in general, and highlight the need for improved baseline survey and impact analyses to be integrated into FFS projects.

## 1. Introduction

Sub-Saharan Africa's (SSA) population, 856 million in 2010, is projected to exceed two billion shortly after 2050. Close to 218 million people, roughly one in four, are currently undernourished, yet African governments spend between just 5% and 10% of their budgets on agriculture, well below the 20% average that Asian governments devoted to the agriculture sector during the Green revolution [1]. While governments in Africa have made a commitment to spend 10% of their budget on agriculture to meet key targets on sustainable development and food and nutritional security, only a few have succeeded in doing so [2].

The focus on how best to address food security issues has shifted over the past decade from one primarily concerned with achieving national food security to one focused on household food security [3]. The challenge of how individual households will in the future sustainably access sufficient, safe and nutritious food to meet their dietary needs and food preferences, focuses attention on the dominant scale at which management decisions are made in developing countries. Individual decisions made by tens of millions of farmers determine the status and trends in productivity and ultimately the sustainability of agricultural systems. Progress towards sustainable solutions requires effective research and extension systems to be able to connect and work with often highly decentralized, isolated and semi-literate populations.

Besides small equipment, fertilizers, high-quality seeds and favourable markets, farmers also need access to new skills and knowledge that will allow them to better manage their resources. However, the past two decades have witnessed weakening support for public extension systems in developing countries in general, including West Africa [4]. As formal extension systems in West Africa have declined, a mosaic of local and national stakeholders in many countries has emerged to fill some of the gaps. The specific mix of actors varies with the country, but generally includes non-governmental organizations (NGOs), farmer organizations and the much-reduced government extension services. Debate continues on how these services should be structured to maximize efficiencies with limited resources and what additional investments are likely to be cost-effective and sustainable [3,5,6].

In this paper, we examine a case study from cotton production systems in Mali. The link between cotton and food security relates to income generation. Food security among small-scale and marginal farmers has been shown to be linked to increased farm incomes, resulting from progressive diversification of small farmers into high-value crops and by increased rural employment through the production and processing of more labour-intensive, high-value crops and value-added products [3].

Cotton is the principal engine of economic development in rural southern Mali, generating benefits to farmers, rural communities, private traders, cotton companies and the national government [7]. In Mali alone, almost four million farmers are engaged in cotton farming, accounting for between 50% and 75% of total export earnings for the country [8]. However, with this economic development has come increasing environmental degradation, in large part owing to the chemical inputs such as pesticides, increased tillage and high nutrient demands of the crop [9]. Evidence from studies in other areas and crops in the West African region shows that risks to human health and environment are inherent in the current use of certain hazardous pesticides by small farmers [10], presenting a challenge to food security, human health and livelihoods.

Current extension approaches can be grouped into four categories: (i) linear or ‘top-down’ transfer of technology; (ii) participatory ‘bottom-up’ approaches; (iii) one-on-one advice or information exchange and (iv) formal, structured education and training [11]. This paper examines the structure and results from the Mali programme in West Africa that uses a participatory- or community-based educational (CBE) approach called the farmer field school (FFS). We present the results of a case study from the cotton sector in Mali in which the use of hazardous insecticides was substantially reduced through a bottom-up approach. We present evidence that CBE investments were effective in substantially reducing use of hazardous pesticides, and show evidence for diffusion of new practices to other farmers.

### (a) Farmer field schools: origins

FFS were developed in the early 1990s in southeast Asia as an alternative to the ‘top-down’ extension methods used during the Green revolution. The FFS approach developed as a response to widespread outbreaks of the brown planthopper (BPH; *Nilaparvata lugens* Stal) in irrigated rice [12–14]. As part of the Green revolution, credit for farmers was linked to obligate purchases of inputs, including pesticides [15]. Extension efforts associated with the Green revolution recommended farmers use calendar-based applications of broad-spectrum insecticides. This was despite the fact that researchers had known for some time that BPH outbreaks were triggered largely by the overuse of insecticides, eliminating beneficial arthropods (predators and parasitoids), and thereby causing an indirect effect known as pesticide-induced resurgence [16–19]. Tropical irrigated rice is especially rich in beneficial species [20,21].

Other consequences of wide-scale use of pesticides have become apparent over time. The past 50 years have seen increasing evidence of insecticide resistance, in which insect pests become unresponsive to certain classes of pesticides as a result of evolutionary pressure and overuse [22]. In Africa, studies raise the possibility that widespread use of insecticides in agricultural systems may be responsible for reduced efficacy of insecticide-impregnated bed nets used in malarial control programmes [23].

Through simple field-cage experiments, observations and discussion, the first FFS, beginning in the 1990s, showed farmers the importance of beneficial arthropods in regulating pest populations. Other exercises demonstrated that rice plants are able to compensate for pest damage to a remarkable degree while in the tillering stage, without suffering yield loss. These two key concepts—existence and importance of beneficial insects, and ability of the plant to compensate for damage caused by pests—were the arguments used to encourage farmers to experiment with easing back on insecticide use on rice, to see for themselves whether they could reduce or eliminate hazardous insecticide use without loss of yield. Biological pest control is today considered an important ‘ecosystem service’ [24,25], the global annual economic value of which has been estimated to be around US$ 4.5 billion [26].

The FFS has much in common in its approach with ‘adaptive management’— a school of thought developed by natural resource scientists and managers in North America who, confronted by not being able to successfully communicate important management messages to policy-makers and the general public, sought a new framework for effective communication and action [27–30]. Natural resource scientists, beginning in the 1980s, recognized the need to develop a common language in which the key ideas from science could be effectively used to engage a broad range of stakeholders to effectively guide collective decisions related to the management of commonly held natural resources. The ideas of adaptive management are central to important global policy guidelines on natural resource management, including the ‘12 Principles of the Ecosystem Approach’ of the Convention on Biodiversity [31]. However, adaptive management has been slower in finding an audience among institutions involved in agricultural development (but see [1,32–34]). The three key concepts of adaptive management underlie FFS programmes: (i) being evidence-based (experimentalist), (ii) focusing on the scale at which management decisions are actually made (place sensitive) and (iii) addressing issues at multiple scales, from local to international (multiscalar) [27].

### (b) The farmer field school programme in West Africa

The FAO West African Regional Integrated Production and Pest Management Programme (IPPM), established in 2001, is currently active in seven countries in West Africa (Benin, Burkina Faso, Guinea, Mali, Mauritania, Niger and Senegal). The programme is based on an FFS approach, working with farming communities and other stakeholders to introduce farmer-based methods for field testing, adapting and adopting improved farming practices. To date, the FAO-IPPM programme has worked with approximately 160 000 farmers through season-long FFS.

An FFS aims to illustrate key concepts through simple experiments in order to develop practical knowledge, skills and improve individual and collective decision-making. The model of a typical FFS has been described in numerous publications [12,35,36] and in box 1. An FFS is intended to be a field laboratory for learning in a low-risk setting for farmers. Inputs are provided by the programme and investment in time made by farmers—one-half day each week, for 12–16 weeks, depending on the crop—is their investment as opportunity cost.

Box 1. **Characteristics of a typical FFS.**

— A group of 20–25 farmers, assisted by a project-trained facilitator, prepares two training plots of around 1000 m² total. The FFS group spends roughly one-half day per week setting up experiments, making observations and jointly managing the two plots, one using local, conventional farming methods and a second plot testing new practices appropriate to the crop and location.
— Exercises are explicitly designed to introduce topics in synchrony with the specific growth stages of the crop, over the course of a cropping season.
— Farmers are asked to summarize their observations with depicting the status of the observed plots, including plants, insects, water levels, weeds, etc. Drawings are an effort to engage less literate farmers.
— Additional ‘special topics’ are introduced over the course of the season to introduce or reinforce key concepts, e.g. demonstrations of pesticide toxicity, soil water-holding capacities, composting methods, etc.
— Exercises include agronomic techniques for planting, soil fertility management, and integrated pest management (IPM), varietal comparisons and marketing.
— At the end of the FFS season an ‘open house day’ is generally held in which other farmers from the community and from adjacent communities are invited, along with local government personnel and civil society to see presentations by FFS farmers and to discuss their outcomes from the season.
— The land used is either donated by the community, rented from a local farmer, or seeds, inputs and labour are provided and proceeds from harvest go to the land owner.

FFS programmes start in a new country by training staff from government extension agencies, non-governmental organizations (NGOs) and farmers from farmer organizations, as ‘facilitators’ to conduct field schools. Season-long training-of-facilitators (ToFs) combines training in non-formal education with practical training in a variety of agronomic topics. Training is split between field activities and classroom and, as with the FFS, follows closely the content and timing of a cropping season. In most programmes, a ‘practice’ FFS is associated with each ToF. A diversity of ToF training models has been developed to reduce time spent by participants and to minimize costs while still maintaining the season-long orientation of the training [37].

Developing FFS for the first time in the country is almost always done by bringing experienced national training staff from other, preferably nearby countries, in which FFS programmes are already underway. This ‘south–south’ collaborative approach is intended to increase cost efficiency and to establish or reinforce regional networks. The first ToF in Africa (Ghana, 1996) used ‘master trainers’ from Vietnam and the Philippines.

Particular emphasis recently has been put on training farmers as facilitators, as they may offer the best avenue for developing a sustainable cadre of skilled workers in the community and at district levels. Training farmers as facilitators offers the possibility of scaling up FFS training that would otherwise be limited, if restricted to training only government agents. To date, approximately 66% of the more than 3200 facilitators trained in the seven countries were farmers, 17% of whom were women.

After the training season, FFS participants are encouraged to form groups and to continue activities in subsequent seasons. In Mali and other programme countries, post-FFS groups visit each other's fields on a periodic basis to discuss their practices and problems. The facilitator is present in his or her capacity as a technical resource person and to provide links to other technical resource persons at district or national scales.

### (c) Strengths and weaknesses of the farmer field school approach

The results from the early FFS programmes were sufficiently positive to encourage countries and donors to invest further in FFS programmes. Twenty years on, FFS projects have been undertaken in an estimated 90 countries, including 30 countries in sub-Saharan Africa [38]. Farmer participatory approaches are rapidly gaining acceptance as effective and sustainable methods for developing more ecological crop and pest management strategies [39].

In measuring impacts from extension efforts, programmes face numerous challenges, chief among them being complexity and scale. Highly decentralized efforts, involving large numbers of farmers managing relatively small plots of land, and influenced by a large number of social, economic and environmental factors usually require detailed and expensive efforts in survey and analysis [4].

A variety of studies have shown FFS to have positive results in terms of increasing farm productivity, improving efficiencies in use of combined chemical and organic fertilizers and substantially reducing hazardous chemical pesticide use [36,37,40–43]. Benefits have been reported related to poverty reduction, improved community organization, farmer empowerment and collective action [40,42,43–45] and in overcoming farmer fears of risks from adopting new technologies [43].

Communities that have been involved in FFS are seen as good entry points for ‘farmer participatory research’ (FPR) [41,46] where researchers work closely with communities to study key problems. The limitation of FPR is the degree of investment of time by experts, yet these investments can result in close-knit researcher–farmer collaboration and yield important benefits to both farmers and the research and development communities [47,48]. An important benefit includes graduating a growing cadre of researchers with strong farmer-collaborative skills and insights on social-science processes.

By contrast, Sherwood *et al.* [49] described an example of attempted FPR in which researchers and politicians, not the farmers, ended up determining the priorities and activities. They conclude that without adequate social-science skills, researchers can unknowingly misuse the farmer–researcher bond. They document the failure to successfully scale up an FFS project in Ecuador. Once the project had finished, national institutes captured control and transformed the people-centred approach into an expert-led, technology-centred approach more in line with the previous ‘top-down’ paradigm (‘elite capture’). They conclude that scaling up FFS programmes beyond a certain ‘niche’ level is inherently risky because it involves an increased concentration of resources and power that is attractive to institutional vested interests.

The concern for sustainability of FFS approaches after the end of projects is expressed by a number of authors [3–5,49,50]. We note that in Mali, during the second phase of the programme (2006–2011), some 30% of the operating budget derived from partner projects that joined together after the start of the FFS programme looking to benefit from the use of the established FFS administrative and technical structure. This may offer a clue to one way in which the issue of sustainability could be addressed—established FFS national units, embedded within national ministries that can act as service providers to a diversity of future projects who seek a well-established and functional infrastructure for working with farmers and communities. At the policy level, the FFS approach in Mali has been inscribed in the national agricultural investment strategy (PNISA) [51], thereby becoming part of the national policy and open to future support by government budgets and by other donors.

Feder *et al.* [50] in a review of the CBE literature also point to ‘elite capture’ (capture of the FFS process by the dominant social group in the community) as a major constraint. They go on to speculate that incentives for collective action within communities (e.g. to join a local farmers group for the purpose of group-based extension) may be strong, because participants expect to benefit directly from their participation. However, participation by farmers in collective actions outside their own communities may not be attractive without some additional incentive beyond that of social capital.

Ortiz *et al.* [42] conclude that FFS participants can benefit from enhanced knowledge and higher productivity in addition to improved community organization. Extension organizations can benefit from experience with participatory methods by seeing examples of how recommendations can be adapted to local environmental conditions. The authors also consider that FFS offers an option for using scarce resources more efficiently.

Bentley *et al.* [41] examined three different extension methods in the control of bacterial wilt (*Ralstonia solanacearum*) on potatoes in Bolivia. FFS were found to be most effective, but community workshops were found to be almost as effective as field schools for teaching most ideas. Radio spots were the least effective. The authors conclude that extension methods should be chosen based on the local context. The more complicated, tedious and counterintuitive the new technology, the more important it is to use an intensive extension method, such as FFS, and the less likely that a mass media approach will be successful.

However, more than just good technical content and participatory approaches are needed to enable successful outcomes. Any extension approach that fails to take into consideration local constraints or investigate priorities with farming communities can ultimately undermine farmers' willingness to adopt new practices. By not involving farmers and other local stakeholders at the project developmental stages, project designers can impose predetermined choices that may not be appropriate for the diversity of farming contexts [52].

Another common critique of FFS programmes is the cost to train farmers and facilitators compounded by an apparent lack of diffusion of knowledge and skills from trained to untrained farmers [35,50,53]. We show in this paper an example in which diffusion of pest control practices apparently took place to a substantial degree.

### (d) Managing risks from pesticides in West African agricultural systems

Jepson *et al.* [10] suggest that three categories of activities are needed in any country to manage risks from practices involving synthetic pesticides: (i) realistic policies for legislation and regulation of pesticides, backed by the political will needed to enforce proper management of pesticides throughout the entire pesticide ‘life cycle’ [54], from import, packaging, labelling and sales, through to use and destruction of empty containers; (ii) development and deployment of cost-effective tools for monitoring and surveillance of farming practices and pesticide environmental concentrations to estimate pesticide risks to human and environmental health; and (iii) end-user education, in which farmers learn of the risks and benefits associated with various practices (risk communication) and are aided in developing alternative management approaches. We focus on this last point by highlighting a case study from Mali that examines the relationship between farmer training and pesticide risk reduction in cotton.

### (e) Cotton in Mali

The cotton sector in Mali has historically been managed by vertically integrated, state-supported cotton companies, with a guaranteed price and market for seed cotton, access to inputs and equipment on credit; with improved varieties developed by the cotton-supported regional research system [7].

Cotton-growing households in Mali have traditionally been some of the most prosperous. The economy of Mali depends mainly on agriculture, and cotton is Mali's most important cash crop. Since 1980, cotton has accounted for 8–9% of gross domestic product (GDP), and from 50% to 75% of export earnings. The cotton sector uses around 3 796 000 farmers in more than 200 000 extended family households, covering an area of approximately 163 000 km [8]. The cotton company registers individual cotton-growing ‘households’, which are most often an extended family unit that can be as many as 15 adult males.

The Malian textile company La Compagnie Malienne de Développement des Textiles (CMDT) was founded in 1975. The CMDT annually provides farmers access to inputs (fertilizers, seeds, pesticides and agricultural equipment) on credit. Inputs are provided prior to the season, and the cost of these inputs is recorded and later deducted from the payment to farmers at harvest. CMDT works closely with a farmer federation, the Union Nationale des Sociétés et Coopératives Producteurs de Coton du Mali. Cotton production under CMDT is highly structured, beginning with four major cotton-producing regions corresponding to CMDT subsidiaries (*filiales*), comprising a total of 41 sectors, divided into 288 communes and, at village level, 7177 Cotton Producer Cooperatives [8].

The cotton sector in West Africa shows parallels with the production systems in Asia in that it has similarly been subject to top-down extension efforts that promoted calendar-based intensive applications of hazardous insecticides. As it was with Asian rice during the Green revolution, credit programmes for cotton farmers in many countries, notably Benin, Ivory Coast and Mali, obliged participating farmers to purchase certain types and quantities of pesticides [15].

The IPPM/FFS programme began activities with the CMDT in 2003, training both technicians from the CMDT and select farmers from the communes in season-long ToF. To date, some 359 facilitators have been trained of which 127 are farmers (called ‘farmer–facilitators’ as distinct from ‘technician–facilitator’). The farmer–facilitator was at that time a new actor in the established cotton extension hierarchy. Today, they do much of the FFS training and advise other farmers in the community. Farmer–facilitators are selected for training based on criteria including literacy, health, willingness and location. The ‘technician–facilitators’ act as technical focal points, providing technical and administrative support to the farmer–facilitators and links to the company administrative hierarchy as well as to the national research and extension services. With this structure, approximately 25 980 cotton farmers in Mali have been trained through FFS.

### (f) Pesticide use in Malian cotton

Before the IPPM programme, three types of pest-control methods were proposed to cotton farmers: (i) calendar treatments, (ii) ‘stage-specific treatment’ or the *Lutte Etagée Ciblée* (LEC) and (iii) threshold sprays. The list of insecticides recommended over the years by the cotton company include a range of chemicals, all registered at the time by the regional authority for pesticides (The Sahelian Pesticide Committee) and authorized for use by the CMDT. The cost per hectare of insecticide treatments ranges from US$ 8.93 for threshold treatments to US$ 71.43 for calendar treatments (table 1).

**Table 1.** Estimated costs per hectare associated with four different insecticide treatment regimes for cotton in the CMDT cotton-growing regions of Mali. LEC, *Lutte Etagée Ciblée* or ‘stage-specific treatment’; IPPM Integrated Production and Pest Management; TS, threshold sprays; CT , calendar treatment. Source [55].

cost of pesticide use by practice (US$ per hectare)			
treatment method			
LEC	IPPM	TS	CT
35.72	1.79	8.93	71.43

Principle pests of cotton in the region include the bollworm complex (e.g. *Helicoverpa armigera*, *Earias biplaga*, *Earias insulana*, *Diparopsis watersi*); leaf-feeding species (e.g. *Syllepte derogate*); aphids (principally, *Aphis gossypii*); pod-sucking insects (*Dysdercus* spp.) and thrips (e.g*. Bemisia tabaci*) [56].

The IPPM/FFS pest management recommendations are based on field scouting by farmers. If signs of eggs, larvae and damage are found, then farmers are encouraged to use biopesticides derived from plant extracts with low mammalian toxicity, the most widely available and popular being neem (*Azadirachta indica*). If a pest problem persists and is not responding to plant-extract treatments after three or four applications, then use of a synthetic insecticide with relatively low mammalian toxicity is recommended.

Neem is widely known for its complex array of insecticidal properties, including at least three modes of action: as an antifeedant, growth regulator and in disrupting digestive enzymes [57]. The published literature shows a number of studies in which neem has been tested and shown effective against a variety of pests of cotton [58–60]. According to our observations and discussions, neem trees are widely but unevenly distributed throughout the region, and the demand for neem as an insecticide has increased to the point where farmers in Mali are beginning to purchase imported neem formulations from international sources. Communities in the cotton sectors of Mali have begun to organize to plant more neem trees.

Some 10 different formulations of extracts from locally available plants have been devised by the IPPM/FFS farmers in Mali. These have not been systematically tested for efficacy or potential risks, but should be. In contrast to neem, some plant extracts are ineffective as insecticides and others are effective, but have high mammalian toxicity (e.g. tobacco). Work needs to be done to ensure that farmers do not unintentionally develop inefficacious or hazardous alternatives.

## 2. Methods

### (a) Study sites

We report on changes in pesticide use in cotton from two of the 41 sectors: the sector of Bla, located approximately 250 km east of the capital of Bamako (12°57′0″ N 5°45′0″ W), and the sector of Bougouni, located 170 km south of Bamako (11°25′0″ N 7°29′0″ W). To date, in Bla, a total of 36 farmer–facilitators have attended ToF training and are active in the field. These farmer–facilitators work across all 56 villages in the six communes of the sector. Approximately, 1461 farmers have attended FFS, out of a total of 4324 cotton households in the sector—a coverage of 34%, assuming one trained FFS farmer per household.

The sector of Bougouni was selected for comparison as it was one of the few sectors in which FFS training had not yet begun during the time period of the study. However, being located 170 km to the south, Bougouni has higher and more consistent rainfall patterns (around 1000–1100 mm) compared with the sector of Bla (800–900 mm). The sector of Bla is on the very northern ecological border of where cotton can reasonably be grown, whereas the sector of Bougouni is in a highly favourable ecological zone for producing cotton. The difference in rainfall regimes between treatment and control sectors is an acknowledged weakness of this study.

### (b) Data gathering

This study was based on a simple gathering of data from the cotton company historical records. The cotton company (CMDT) maintains records at sector-level facilities, which gather information from multiple communes. Cotton cooperatives exist in almost every village and are responsible for gathering data from the households in their village (e.g. how many hectares of cotton the household plans to plant the following season) and communicating this information to the sector. The sector-level office records seasonal distributions of inputs, provided on credit, to individual households, and acts as point of sale for the cotton harvest at the end of the season, at which time farmers are required to pay back their loans. Records are kept on yield, price paid for cotton at harvest and the amounts and cost of inputs provided, including fertilizer and insecticides.

Prior to each season, the cotton company estimates the number of hectares of cotton that will likely be under cultivation based on farmer cooperative estimates. The quantity of pesticide needed by farmers is calculated as the number of hectares anticipated to be under cultivation multiplied by the recommended number of litres of insecticide. The volume rate recommended by the cotton company is 1 l ha^−1^, four times per season for Bla and six times per season for Bougouni. The difference in rainfall may be responsible for greater pest pressures, real or perceived; hence, the recommended higher treatment volumes.

Company records from commune offices from 2003 to 2010 allowed a calculation of farmers' willingness to purchase the insecticides provided for sale by the cotton company. From the difference of what was available and recommended for purchase, and what was actually purchased by the households, we calculated the simple metric ‘per cent of recommended pesticides purchased’. Because the company data are aggregated at the commune level, the independent unit of analysis is average pesticide purchased per hectare, per commune. There were six communes in Bla and four communes in Bougouni. Data for all 4324 households in Bla were recorded for analysis. In Bougouni, a sample of 800 farmers was taken from the company records (every tenth record).

In our analysis, we focus on two recorded measures: pesticide purchases and yields over time. The data are aggregated at the commune level, so the unit of analysis is a commune (56 villages comprise six communes in Bla, and 27 villages comprise four communes in Bougouni).

### (c) Statistics

We used a linear mixed-effects model [61] in order to investigate the relationship between farmer training and pesticide use over time (repeated measures) for the two sectors of Bla (treatment) and Bougouni (control). The metric ‘per cent of recommended pesticides purchased’ was the dependent variable. The measurement unit ‘commune’ was set as a random effects, independent variable, with ‘year’ and ‘sector’ (equivalent to ‘trained farmers’ versus ‘untrained farmers’) considered fixed independent variables. We fit the same model a second time, comparing farmer training and yields over time.

Visual inspection of residuals plotted against fitted values showed no patterns, fulfilling the requirement of homogeneous residuals.

## 3. Results

### (a) Pesticides and yields

**Figure 1.**
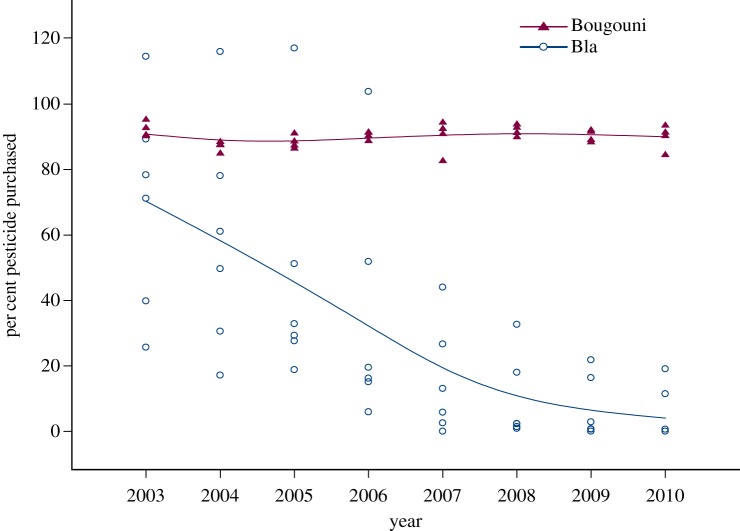
Percentage of pesticides purchased. Unit of measure is the total amount of pesticide purchased by farmers divided by the total volume of pesticide made available by the cotton company for that commune: a total of six communes of the sector of Bla and four communes of the control sector of Bougouni. Projections for pesticide volumes to be provided to farmers for sale by the cotton company are based on total surface area anticipated to be under cotton production for the coming season, multiplied by the number of litres of insecticide recommended per hectare (4 l ha^−1^ for Bla and 6 l ha^−1^ for Bougouni). Significant differences exist between means for the two sectors, *p* < 0.05, for all years except 2003 and 2004. (Online version in colour.)

Between 2003 and 2010, pesticide use in the sector of Bla fell by 92.5% for all six communes, with 1461 (34%) of the 4324 of the cotton-farming households in the sector having received training in IPM through FFS by 2010. In the sector of Bougouni, where no FFS training had taken place, pesticide use was unchanged over the same period (figure 1).

**Figure 2.**
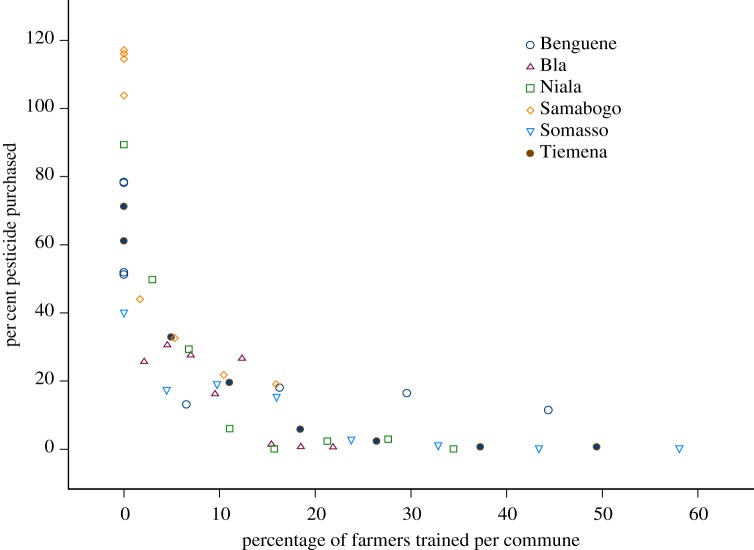
Percentage of pesticides purchased by percentage households trained in FFS. Percentage of pesticides purchased by farmers by percentage of cotton households in commune trained in FFS for the six communes of the sector of Bla. The curvilinear relationship indicates possible diffusion of practices from FFS-trained to non-trained farmers in the six communes. (Online version in colour.)

Plotting the per cent of recommended pesticides purchased, against the per cent of households trained in FFS, shows a steep decline in pesticide purchase that strongly suggests a high correlation with the per cent of households in the commune trained (figure 2). Only the first 2 years, 2003 and 2004, showed no significant differences in pesticide purchased between the two sectors.

**Figure 3.**
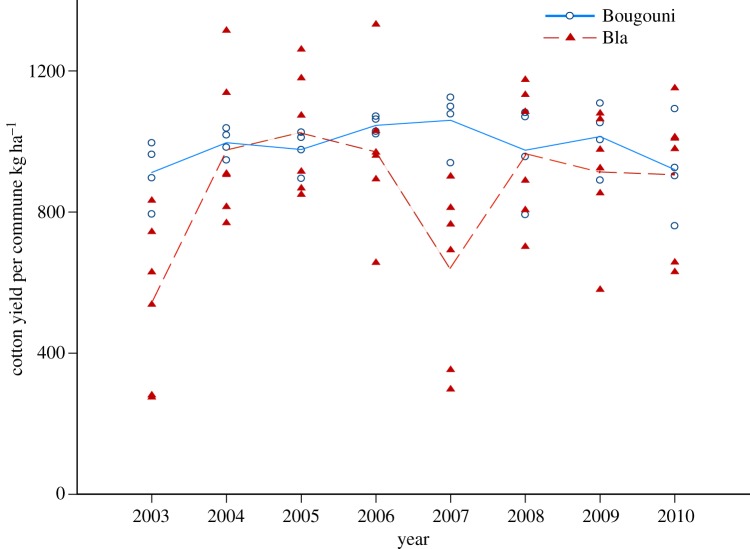
Cotton yields by year. Cotton yields in the cotton sector of Bla vary substantially year-to-year, compared with Bougouni, most likely due to variability in rainfall. There are no apparent trends in yields over time.

Cotton yields in the region show high variability over time (figure 3) [62], more so for the sector of Bla. We attribute this to the decreased average rainfall and increasing rainfall variability as you move north towards the Sahara desert. There is no apparent shift in yield patterns for the sector of Bla, which might be expected if the shift in pesticide use was to the detriment of the crop. Except for 2007, the period of time between 2004 and 2010 shows no difference in average yields between the two sectors.

### (b) Economic costs and benefits

The median value of insecticides spent by farmers, as the percentage of gross revenue (value of harvest) over the 8 year period, was 2.7% for the sector of Bla and 14.2% for the control sector of Bougouni. Starting with the 2003 pesticide use figures as a baseline (100%), cotton farmers in the Bla sector saved approximately 47 000 l of synthetic insecticide, worth in the order of US $ 470 000. Farmers in Bla spent an estimated US $ 84 000 on neem treatments at an average cost of US $ 1.8 per treatment and assuming an equal number of treatments to those farmers in Bougouni. The shift from synthetic to biopesticides by the farmers in Bla, therefore, translates into a saving of approximately US$ 386 000 over the 8-year period.

At the national level, the average cost for an FFS in Mali is approximately US$ 600, or US$ 24 per farmer. The total cost to train the 1461 farmers in Bla was around US$ 35 000. A much more conservative estimate of the cost to train one farmer is to calculate an average cost based on all programme costs divided by the total number of farmers trained by the programme, which is approximately US$ 84 per farmer. With this estimate, the total cost of training all farmers trained in Bla is approximately US$ 122 724. In either case, the cost to train farmers in FFS was compensated by the total pesticide costs savings, even in the more conservative case by a factor of 3 to 1.

### (c) Economic and non-economic benefits

We estimated a benefit to farmers of around US$ 386 000 from savings in pesticide costs. However, to assess the larger scope of non-economic benefits is more challenging. The benefits to the environment and human health from not applying the recommended additional 47 000 l of hazardous insecticides are easy to imagine but difficult to measure. To this end, the programme has been working in collaboration with Oregon State University's Integrated Plant Protection Center and other partners in the region, to build capacity for analysis of pesticides in the environment [63]. Related work with Oregon State University has involved adaptation of cutting-edge models to estimate probable impacts of pesticide use on a broad range of biotic and human health indicators. The data emerging from other production systems in the project countries strongly suggest that there are substantial risks posed to human health and key environmental indicators from current pesticide-use practices in the region [10]. This work was supported under a project focused on the Senegal and Niger River basins, and has not yet been undertaken for the cotton areas.

## 4. Discussion

### (a) Adoption of new practices through farmer-to-farmer diffusion

In the FFS-trained sector of Bla, the correlation between FFS training and pesticide purchases shows a steeper drop than expected had the correlation between farmers trained and pesticides purchased been strictly linear. These data suggest that diffusion—where practices adopted by farmers as a result of training are also adopted by other farmers in the community—is likely to be taking place to a significant extent.

Diffusion would of course be a highly desirable outcome for any FFS effort if new practices are to be scaled up to reach large numbers of farmers in the shortest possible time. But, this poses something of a paradox: if FFS activities over the course of a season are needed to advance farmer understanding to a point where farmers feel confident to take the risk of not following previous recommendations, then what motivates other farmers to follow suit, who have not undergone season-long FFS training?

The question of diffusion in FFS has been a subject of discussion in the literature [4,35,36,41,49,64,65]. Diffusion is considered by some authors to be a prerequisite for any CBE effort to be considered a cost-effective approach [66]. Witt *et al.* [67], looking at data from a 2006 external review of the first phase West African IPPM/FFS programme, calculated that diffusion was not evident at that time and suggested that small numbers of farmers, widely dispersed over large geographical areas, represented an insufficient ‘critical mass’ of farmers in any one location to induce change beyond the participants themselves. One recommendation by the evaluators, besides training more farmers, was to begin to strategically cluster FFS to take advantage of building local networks of trained farmers among nearby communities.

We speculate that there are several reasons why this cotton sector in Mali might be witnessing diffusion in the adoption of new practices aimed at reducing hazardous pesticide use:

(i) *A high proportion of cotton-farming households having undertaken FFS training*. Likely the most important factor, the percentage of farmers trained in the sector was quite high at 34%; however, the shape of the relationship suggests that the reduction in use was attained after approximately 20% of the households had received training (figure 2). It will be important to see whether similar patterns emerge elsewhere, in locations where FFS training attains similar proportions of a local population.
(ii) *A simple technology*. The technology is relatively simple to formulate with locally available materials, and the concepts remain largely familiar (scout for presence of pests at an early stage of the crop, and apply biopesticides if found).
(iii) *Lower costs*. Cotton is a high-value cash crop for which significant resources have historically been expended by farmers for purchase of pesticides. IPPM treatment costs per hectare compared with other treatment methods ranged from 2% compared with cost per hectare of calendar treatments, to approximately 20% compared with the estimated cost per hectare of threshold treatment methods. This does not include time spent preparing the formulations for locally prepared biopesticides (table 1).
(iv) *Low mammalian toxicity and high efficacy*. Studies undertaken elsewhere to look at the efficacy of neem in controlling cotton pests show good efficacy and duration of effects in the field [58–60]. This programme has not undertaken such studies, so can only look at what the farmers say and the data. Speaking with local farmers in Bla, project staff are told that neem performs well and that it is rare that they encounter a situation where they are obliged to use a synthetic insecticide. In terms of yield, despite the high spatial and temporal variability, the data show no apparent reductions in yield.
(v) *Increased social capital*. In 2010, the farmer–facilitators in Bla organized and took on an additional role as field scouts to evaluate the status of pest populations in their communities and adjacent communities. Their goal was to carry out periodic transect surveys at critical times during the cotton season; at the same time speaking with farmers along their route, who scout their own fields and have observations to contribute. From this, pest status reports are transmitted on local rural radio. In order to carry out these tasks, the facilitator scouts were motivated to secure a microcredit loan from a local bank to purchase motorcycles and fuel. The loan was agreed to be reimbursed by the contributions of cotton farmers in the sector through the cotton cooperatives. This represents a positive step towards independence of the FFS farmer facilitators and a measurable indicator of group initiative and community support and cohesion above the village level.
(vi) *Rural communications*. Over the past 5 years, 25 sessions related to FFS issues and outcomes in cotton were aired on rural radio, the majority in local languages. National television is frequently present for FFS ‘open houses’ and several 10–15 min films have been produced on the FFS programme in a variety of cropping systems. We support the idea from other authors who have recommended that FFS projects need to develop an integrated approach that includes video, rural radio and other mass communication methods [6,65].

Taken as a whole, the factors listed offer a compelling argument, supported by the data, to suggest that, with time, diffusion of the adoption of improved pest-control methods from FFS to non-FFS farmers can take place. Further work needs to be done with the assistance of sociologists, economists and agronomists, to examine this and other case studies more closely.

### (b) Impact analyses

The number of impact studies examining outcomes from FFS and other CBE efforts are increasing over time and good case studies can be found in the literature [40,41,44,67–69], as well as cogent and informative critiques at a meta level [3–5,35,50].

A common weakness of FFS projects is that data do not allow the definition of good counterfactual scenarios, because no control area was available or only insufficient baseline data existed [68]. The data in our study, provided by the cotton company, were a fortuitous and unusual opportunity to access data, from over an 8-year period, which did not require a large investment in time or resources and did not depend upon farmer recall. These data look only at two factors: pesticide purchases and yields. While we feel the study, within its limited scope, is compelling, we recognize the study itself does not substitute for a more formal and in-depth impact study as it does not, for example, provide an analysis of social, economic and environmental changes owing to FFS training.

In-depth impact assessments can be costly and technically demanding. Stronger partnerships between research organizations and development agencies can help develop the human technical capacities needed. The current, long-term partnership on the IPPM/FFS programme with Oregon State University has resulted in socioeconomic and environmental monitoring methods, reported elsewhere in this issue [10,63], being built into the design of current and future FFS projects to enable tracking and measurement of outcomes.

Projects need to conduct high-quality baseline studies, built in from the beginning and using at least a minimal set of social, economic, agronomic and environmental measurable indicators that provide suitable metrics of practices, production, costs and benefits, but also measures of important changes in key social and environmental factors. Recognizing that the most informative measures of outcome will likely only be able to be determined after the end of a project, these data should be put into the public domain in some form of open-source database for use by others in the future. Such a database is currently under construction by the Oregon State University/FAO programme.

Building better baseline surveys and follow-up impact studies into FFS programmes will require that donors are fully on board and agree to provide requisite support for thorough efforts at gathering, managing and analysing data; followed by appropriate synthesis and recapitulation back to the participating communities. The successful scaling up of high-quality, community-based approaches cannot be based on small-scale, short-term projects, but needs to be conceived of on decadal and regional scales.

## 5. Conclusion

The data from Mali show a marked reduction over an 8 year period in the use of hazardous insecticides by more than 4324 cotton-growing households. With roughly 20% of these households involved progressively over time in FFS training, hazardous insecticide use fell by 92.5% for all cotton-growing households in the sector. By contrast, pesticide use was unchanged over time in the sector with no farmer training taking place.

FFS activities help advance farmer understanding in a low-risk, peer-group setting. The process of adaptation and adoption begin when farmers feel confident to take the risks of experimenting with and evaluating new methods in their own fields. We conjecture that diffusion of improved pest-management practices from FFS to non-FFS farmers may likely have occurred, when a low-cost, simple technology, providing lower health risks and demonstrated economic benefits was successfully used by an increasing and substantial proportion of farmers in the sector.

Historically, centralized ‘top-down’ extension systems did not meet the challenges of agriculture in developing countries. They were expensive and cumbersome, and therefore were not sustainable and did not persist. However, the alternative of being ‘participatory’ is no guarantee of success. The literature suggests that weaknesses in extension efforts, including FFS projects, most often result from project designs not closely involving stakeholders from the beginning, and not taking into account local constraints and priorities.

The complexity and scope of the challenges of agricultural extension are enormous. We believe that an adaptive management approach is the best way forward. Failed efforts are bound to occur. The key to enable sustainable progress is to build reflective processes at all scales to enable learning and adaptation from mistakes and successes. Building better baseline surveys and impact studies into FFS programmes will help provide more useful measures of progress and quality. Increased partnerships between national and international universities, NGOs, research organizations and development organizations can aid greatly in this effort by providing the new ideas, skills and human resources needed.

Agricultural extension in developing countries can no longer be usefully looked at as a centralized, monolithic infrastructure. Rather, farmer extension is better seen as a process that involves a diverse mix of actors, beginning with the human resources and infrastructure a district, country or region has at hand. The role of an FFS programme is not to substitute for the extension systems of the past, but rather to facilitate partnerships among the diverse and active mix of actors at all levels in order to collectively develop a dynamic and interconnected extension community, beginning with farmers and building extension from the bottom up.

## References

[1] United Nations Development Programme. Africa Human Development Report. 2012 Towards a food secure future. New York, NY: United Nations Development Programme (UNDP).

[2] Shenggen F Mogues T Benin S 2009 Setting priorities for public spending for agricultural and rural development in Africa. IFPRI Policy Brief. Report no. 12 Washington, DC: International Food Policy Research Institute (IFPRI).

[3] Swanson BE 2008 Global review of good agricultual extension and advisory service practices. Rome, Italy: Food and Agriculture Organization of the United Nations, Research and Extension Division.

[4] Anderson JR Feder G 2004 Agricultural extension: good intentions and hard realities. World Bank Res. Observer 19, 41–60. (10.1093/wbro/lkh013)

[5] Swanson BE Rajalahti R 2010 Strengthening agricultural extension and advisory systems: procedures for assessing, transforming, and evaluating extension systems. Agriculture and Rural Development Discussion paper 45 Washington, DC: The World Bank.

[6] Bentley JW 2009 Impact of IPM extension for small-holder farmers in the tropics. In Integrated pest management: dissemination and impact (eds Peshin R Dhawan AK), pp. 333–346. New York, NY: Springer.

[7] Teft J 2004 Building on successes in African agriculture: Mali's White Revolution: smallholder cotton from 1960 to 2003. Focus 12, 2.

[8] CMDT. 2008 Statement of Mali at the 67th plenary meeting of the ICAC. Ouagadougou, Burkina Faso: Republic of Mali, Office of the Prime Minister.

[9] Moseley WG 2005 Global cotton and local environmental management: the political ecology of rich and poor farmers in southern Mali. Geograph. J. 171, 36–55. (10.1111/j.1475-4959.2005.00148.x)

[10] Jepson PC Guzy M Blaustein K Sow M Sarr M Mineau P Kegley S 2014 Measuring pesticide ecological and health risks in West African agriculture to establish an enabling environment for sustainable intensification. Phil. Trans. R. Soc. B 369, 20130491 (10.1098/rstb.2013.0491) 24535399 PMC3928896

[11] Carson A Morrison S Mulholland M Matthews D Munoz C 2010 Harnessing scientific developments in practice. Belfast, Ireland: College of Agriculture, Food and Rural Enterprise, Agri-Food And BioSciences Institute.

[12] Dilts R 2001 Scaling up the IPM movement. LEISA Mag. 17, 18–21.

[13] Roling NG van de Fliert E 1998 Introducing integrated pest management in rice in Indonesia: a pioneering attempt to facilitate large-scale change. In Facilitating sustainable agriculture (eds Roling NG Wagemakers MAE). Cambridge, UK: Cambridge University Press.

[14] Gallagher KD Ooi PAC Kenmore PE 2009 Impact of IPM programs in Asian agriculture. In Integrated pest management: dissemination and impact (eds Peshin R Dhawan AK), pp. 347–358. New York, NY: Springer.

[15] Fleischer G Waibel H 2003 Pesticide policy and integrated pest management. In Integrated pest management in the global arena (eds Maredia KM Dakouo D Mota-Sanchez D), pp. 49–64. Wallingford, UK: CAB International.

[16] Heinrichs E Mochida O 1984 From secondary to major pest status: the case of insecticide induced rice brown planthopper, *Nilaparvata lugens*, resurgence. Protect. Ecol. 7, 201–218.

[17] Heinrichs E (ed.) 1994 Biology and management of rice insects. New Delhi, India: Wiley East Ltd.

[18] Kenmore P 1980 Ecology and outbreaks of a tropical insect pest of the Green Revolution, the rice brown planthopper, *Nilaparvata lugens* (Stal). PhD thesis, University of California, Berkeley, USA.

[19] Kenmore P Carino F Perez G Dyck V 1984 Population regulation of the rice brown plant hopper *Nilaparvata lugens* (Stal) within rice fields in the Philippines. J. Plant Protect. Trop. 1, 19–38.

[20] Settle WH Ariawan E Astuti T Cahyana W Hakim AL Hindayana D Lestari AS 1996 Managing tropical rice pests through conservation of generalist natural enemies and alternate prey. Ecology 77, 1975–1988. (10.2307/2265694)

[21] Heong K Schoenly K 1998 Impact of insecticides on herbivore-natural enemy communities in tropical rice ecosystems. In Pesticides and beneficial organisms (eds Haskell P McEwen P), pp. 281–403. Dordrecht, The Netherlands: Kluwer.

[22] Head G Savinelli C 2008 Adapting insect resistance management programs to local needs. In Insect resistance management: biology, economics and prediction (ed. Onstad D), p. 305 Amsterdam, The Netherlands: Elsevier.

[23] Diabate A 2002 The role of agricultural use of insecticides in resistance to pyrethroids in *Anopheles gambiae* S.L. in Burkina Faso. Am. Soc. Trop. Med. Hyg. 67, 617–622. 10.4269/ajtmh.2002.67.617 12518852

[24] Millennium Ecosystem Assessment. 2005 Ecosystems and human well-being: synthesis. Washington, DC: Island Press.

[25] Persson L Arvidson A Lannerstad M Lindskog H Morrissey T Nilsson L Noel S Senyagwa J 2010 Impacts of pollution on ecosystem services for the millennium development goals. Project report. Stockholm, Sweden: Stockholm Environment Institute (SEI).

[26] Power A 2010 Ecosystem services and agriculture: tradeoffs and synergies. Phil. Trans. R. Soc. B 365, 2959–2971. (10.1098/rstb.2010.0143) 20713396 PMC2935121

[27] Norton B 2005 Sustainability: a philosophy of adaptive ecosystem management. Chicago, IL: University of Chicago Press.

[28] Holling CS 1978 Adaptive environmental assessment and management. London, UK: Wiley and Sons.

[29] Lee KN 1993 Compass and gyroscope. Washington, DC: Island Press.

[30] Gunderson LH Holling CS Light SS 1995 Barriers and bridges to the renewal of ecosystems and institutions. New York, NY: Columbia University Press.

[31] Convention on Biodiversity. 2000 Ecosystem approach. *Convention on Biodiversity, 15–26 May 2000, Nairobi, Kenya*.

[32] The Prince's Charities. 2011 What price resilience? Towards sustainable and secure food systems. London, UK: The Prince's Charities’ International Sustainability Unit.

[33] Sanginga PC Waters-Bayer A Kaaria S Njuki J Wettasinha C (eds) 2009 Innovation Africa: enriching farmers’ livelihoods. London, UK: Earthscan.

[34] National Research Council of the National Academies. 2010 Toward sustainable agricultural systems in the 21st century. Washington, DC: National Academy of Sciences.

[35] Braun A Jiggins J Röling N van den Berg H Snijders P 2005 A global survey and review of farmer field school experiences report. Nairobi, Kenya: International Livestock Research Institute (ILRI).

[36] van den Berg H Jiggins J 2007 Investing in farmers: the impacts of farmer field schools in relation to integrated pest management. World Dev. 35, 663–686. (10.1016/j.worlddev.2006.05.004)

[37] Settle W Hama Garba M 2011 Sustainable crop production intensification in the Senegal and Niger River basins of francophone West Africa. Int. J. Agric. Sustain. 9, 171–185. (10.3763/ijas.2010.0559)

[38] International Initiative for Impact Evaluation. 2010 Farmer field schools for improving farming practices and farmer outcomes in low- and middle-income countries: a systematic review. Washington, DC: International Initiative for Impact Evaluation (3ie).

[39] Dent D Holderness M Vos J 2003 Integrated pest management at CAB International. In Integrated pest management in the global arena (eds Maredia K Dakouo D Mota-Sanchez D), pp. 493–499. Egham, UK: CAB International.

[40] Davis K Nkonya E Kato E Mekonnen DA Odendo M Miiro R Nkuba J 2011 Impact of farmer field schools on agricultural productivity and poverty in East Africa. World Dev. 40, 402–413. (10.1016/j.worlddev.2011.05.019)

[41] Bentley JW Barea O Priou S Hermeregildo E Thiele G 2007 Comparing farmer field schools, community workshops and radio: teaching Bolivian farmers about Bacterial Wilt of potato. J. Int. Agric. Extension Educ. 14, 45–61. (10.5191/jiaee.2007.14304)

[42] Ortiz O Garrett KA Heath JJ Orrego R Nelson RJ 2004 Management of potato late blight in the Andean Highlands: evaluating the benefits of farmer participatory research and farmer field schools. Plant Dis. 88, 565–571. (10.1094/PDIS.2004.88.5.565) 30812665

[43] Palis F 2006 The role of culture in farmer learning and technology adoption: a case study of farmer field schools among rice farmers in central Luzon, Philippines. Agric. Hum. Values 23, 491–500. (10.1007/s10460-006-9012-6)

[44] Duveskog D Friis-Hansen E Taylor EW 2011 Farmer field schools in rural Kenya: a transformative learning experience. J. Dev. Stud. 47, 1529–1544. (10.1080/00220388.2011.561328)

[45] Friis-Hansen E Duveskog D Taylor E 2012 Less noise in the household: the impact of farmer field schools on gender relations. J. Res. Peace Gender Dev. 2, 44–55.

[46] Krupnik TJ Shennan C Settle WH Demont M Ndiaye AB Rodenburg J 2012 Improving irrigated rice production in the Senegal River Valley through experiential learning and innovation. Agric. Syst. 109, 101–112. (10.1016/j.agsy.2012.01.008)

[47] Nederlof ES Tossou R Sakyi-Dawson O Kossou DK 2004 Grounding agricultural research in resource-poor farmers’ needs: a comparative analysis of diagnostic studies in Ghana and Benin. Wagen. J. Life Sci. 52, 421–442. (10.1016/S1573-5214(04)80024-1)

[48] Roling N Jounkonnou D Offei S Tossou R Van Huis A 2004 Linking science and farmers’ innovative capacity: diagnostic studies from Ghana and Benin. Wagen. J. Life Sci. 52, 211–235. (10.1016/S1573-5214(04)80015-0)

[49] Sherwood S Schut M Leeuwis C 2012 Learning in the social wild: farmers field school and politics of agricultural science and development in Ecuador. In Adaptive collaborative approaches in natural resource governance (eds Ojha HR Hall A Sulaiman VR), p. 334 London, UK: Routledge.

[50] Feder G Anderson JR Birner R Klaus-Deininger R 2010 *Promises and realities of community-based agricultural extension*. Discussion Paper no. 00959 Washington, DC: IFPRI.

[51] Ministere de l'Agriculture. 2009 Programme National d'Investissement dans le Secteur Agricole (PNISA). Bamako, Mali: Ministere de l'Agriculture, Cellule de Planification et de Statistique.

[52] Nederlof ES Dangbégnon C 2007 Lessons for farmer-oriented research: experiences from a West African soil fertility management project. Agric. Hum. Values 24, 369–387. (10.1007/s10460-007-9066-0)

[53] Yang P Shan X Li P Zhou J Lu J Li Y 2008 Effects of training on acquisition of pest management knowledge and skills by small vegetable farmers. Crop Protect. 27, 1504–1510. (10.1016/j.cropro.2008.07.013)

[54] Food and Agriculture Organization of the UN (FAO). 2010 International code of conduct on the distribution and use of pesticides. Rome, Italy: The Inter-Organisation Programme for the Sound Management of Chemicals (IOMC).

[55] FAO IPPM. 2008 Etat de lieux dans le cadre de l’évaluation d l'approche GIPD dans la production de coton et de son extension au Mali. Bamako, Mali: FAO.

[56] PR-PICA. 2010 Rapport de synthese de la troisieme reunion bilan du Programme Regional de Protection Integree du Cotonnier en Afrique (PR-PICA). Ouagadougou, Burkina Faso: PR-PICA.

[57] Schoonhoven LM van Loon JJA Dicke M 2005 Insect–plant biology, 2nd edn Oxford, UK: Oxford University Press.

[58] Reddy T Sannaveerappanava V 2011 Bio-efficiency of commercial neem products and Ha NPV against bollworms of cotton. Int. J. Farm Sci. 1, 105–110.

[59] Nboyine J Abudulai M Opare-Atakora D 2013 Field efficacy of neem (Azadirachta indica A. Juss) based biopesticides for the management of insect pests of cotton in Northern Ghana. J Exp. Biol. Agric. Sci. 1, 321–327.

[60] Khattak M Rashid M Hussain S Islam T 2006 Comparative effect of neem (*Azadirachta indica* A. Juss) oil, neem seed water extract and Baythroid TM against whitefly, jassids and thrips on cotton. Pakistan Entomol. 28, 31–37.

[61] Splus. S-PLUS. 2000 Professional Edition for Windows. Release 3 Splus.

[62] African Agency for Trade and Development (GLOCAL). 2011 An overview of the West and central African cotton sectors, by the actors. African Trade Dev. J. March/April 2011. Report no.: 4.

[63] Anderson KA Seck D Hobbie KA Traore AN McCartney MA Ndaye A Forsberg ND Haigh TA Sower GJ 2014 Passive sampling devices enable capacity building and characterization of bioavailable pesticide along the Niger, Senegal and Bani Rivers of Africa. Phil. Trans. R. Soc. B 369, 20130110 (10.1098/rstb.2013.0110) 24535398 PMC3928895

[64] Duveskog D 2006 Theoretical perspectives of the learning process in farmer field schools. Working paper Rome, Italy: Food and Agriculture Organization of the United Nations.

[65] Bentley JW 2009 Commentary: the right message and method. Int. J. Agric. Sustain. 7, 79–80. (10.3763/ijas.2009.c5005)

[66] Feder G Murgai R Quizon JB 2004 The acquisition and diffusion of knowledge: the case of pest management training in farmer field schools, Indonesia. J. Agric. Econ. 55, 217–239. (10.1111/j.1477-9552.2004.tb00094.x)

[67] Witt R Pemsl DE Waibel H 2008 The farmer field school in Senegal: does training intensity affect the diffusion of information? J. Int. Agric. Extension Educ. 15, 47–60.

[68] Praneetvatakul S Waibel H 2006 Farm level and environmental impacts of farmer field schools in Thailand. Working Paper Hannover, Germany: University of Hannover, Development and Agricultural Economics Faculty of Economics and Management Report no.: 2006 No. 7.

[69] Zuger R 2004 Impact assessment farmer field schools in Cajamarca, Peru: an economic evaluation. Social Sciences Working Paper Lima: International Potato Center Report no.: 2004-1.

